# Bisphenol AF promotes estrogen receptor-positive breast cancer cell proliferation through amphiregulin-mediated crosstalk with receptor tyrosine kinase signaling

**DOI:** 10.1371/journal.pone.0216469

**Published:** 2019-05-06

**Authors:** Qingxia Zhao, Erin W. Howard, Amanda B. Parris, Zhikun Ma, Ying Xing, Xiaohe Yang

**Affiliations:** 1 Julius L. Chambers Biomedical/Biotechnology Research Institute, Department of Biological and Biomedical Sciences, North Carolina Central University, North Carolina Research Campus, Kannapolis, North Carolina, United States of America; 2 Basic Medical College of Zhengzhou University, Zhengzhou, Henan, P.R. China; Universita degli Studi della Campania Luigi Vanvitelli, ITALY

## Abstract

Exposure to bisphenol A (BPA), an endocrine-disrupting compound, is associated with increased risk of estrogen-related diseases, including estrogen receptor-positive (ER^+^) breast cancer. Although bisphenol analogs, i.e. bisphenol AF (BPAF), have replaced BPA in industrial settings, increasing data indicate that these alternatives may have similar or even more potent estrogenic effects. As such, BPAF exhibits increased ER binding affinities than BPA in biochemical assays. However, preclinical studies exploring the effects of BPAF on ER^+^ breast cancer are missing mechanistic data. Thus, we aimed to characterize the effects of BPAF on MCF-7 and T47D ER^+^ breast cancer cells with mechanistic insight. We found that BPAF promoted cell growth and cell cycle progression concurrently with BPAF-induced ERα transcriptional activity and ER-RTK signaling activation. ER signaling blockage revealed that BPAF-induced cell proliferation and ER-RTK crosstalk were ER-dependent. Gene expression data demonstrated that *AREG* is a sensitive target of BPAF in our *in vitro* models. Importantly, we determined that *AREG* upregulation is necessary for BPAF-induced cellular responses. Ultimately, our novel finding that AREG mediates BPAF-induced ER-RTK crosstalk in ER^+^ breast cancer cells supports future studies to characterize the impact of BPAF on human ER^+^ breast cancer risk and to assess the safety profile of BPAF.

## Introduction

Exposure to environmental hormone disruptors, including bisphenol A (BPA), is a major public health concern due to deleterious effects on human health. BPA was a key component of polycarbonate plastics used for everyday items, including plastic bottles and food packaging; however, reports have classified BPA as an endocrine-disrupting compound (EDC) with estrogen receptor (ER) agonist activities. Consequently, BPA has been restricted from many household products due to substantial evidence that BPA elicits adverse effects on human health [1–5]. Particularly, BPA has been shown to promote estrogen-related diseases, like ER^+^ breast cancer, in preclinical animal models [6–9]. Despite efforts to replace BPA with other bisphenol analogs, such as bisphenol AF (BPAF), increasing data indicate that alternative bisphenols may have similar or even more potent estrogenic effects than BPA.

BPAF is a widely used BPA alternative in industrial settings for manufacturing plastics and epoxy resins, as well as in hoses and gaskets on food processing machines [10]. Similar in structure to BPA, BPAF exhibits increased binding affinities for ERα, ERβ, and GPER than BPA in biochemical assays [11–13]. Kitamura *et al*. also reported that BPAF (EC_50_ = 0.05 μM) has more potent estrogenic activity than BPA (EC_50_ = 0.63 μM) in MCF-7 ER^+^ breast cancer cells, indicated by increased ER/estrogen response element (ERE)-mediated transcriptional activity [14]. Similar results were reported by others [11, 12, 15–17], as well as BPAF-induced ER-targeted genes, including *TFF1*, *GREB*, and *CTSD* [18]. BPAF also has demonstrated neurotoxic effects *in vitro* [19] and uterotrophic effects in rats [20]. In zebrafish, BPAF (1–1.5 mg/L) was found to delay the hatching time of exposed embryos [21]. BPAF (50–100 μg/mL) also impeded the maturation of cultured mouse oocytes [22]. Higher concentrations of BPAF (50–200 mg/kg/day for 14 days) were found to induce hormonal antagonistic effects *in vivo*, indicated by decreased serum testosterone levels and testicular *ESR1* mRNA levels in male Sprague-Dawley rats [23]. Collectively, BPAF-mediated estrogenic effects may also have a significant impact on ER^+^ breast cancer risk, which warrants further investigation.

Importantly, BPAF has been detected in the environment, including water sources and soil near industrial plants [24–26]. As such, environmental bioaccumulation of BPAF is an increasing concern because BPAF is estimated to have a 4.8-fold longer half-life than BPA in water, soil, and sediment [26]. Particularly, BPAF has been detected in human urine samples [27, 28], and BPAF exposure levels are expected to rise as it replaces BPA in industrial applications [24, 29]. Therefore, evidence demonstrating the estrogenic properties of BPAF in human cell lines and preclinical animal models merits a comprehensive evaluation of the toxicological and biological consequences of BPAF exposure. Nevertheless, data are limited regarding potential health risks linked to BPAF exposure, including the association between BPAF exposure and ER^+^ breast cancer risk.

Signaling interactions between ER and receptor tyrosine kinase (RTK) pathways are major factors in ER^+^ breast cancer development/progression. Particularly, RTKs, including the EGFR/ErbB family (EGFR, ErbB2/Her2, ErbB3, ErbB4), can activate PI3K/Akt and MAPK/Erk pathways, which can in turn activate Src3/AIB1, an ERα coactivator [30, 31]. Moreover, ER activation can promote the expression of EGFR/ErbB growth factors and their ligands, including TGFα, IGF1, and NRG. Given the bidirectional activation of these signaling networks, ER-RTK/ErbB signaling crosstalk can potentiate ER target gene transcription and cellular responses, including cell proliferation, survival, and invasion [32, 33]. Previously, we reported that phytoestrogen/genistein exposure promoted ER-ErbB2/RTK crosstalk, which mediated genistein-induced ER^+^/ErbB2-overexpressing breast cancer cell growth [34]. ER-RTK crosstalk also contributes ER/RTK-targeted therapeutic resistance due to the activation of these compensatory oncogenic pathways [35, 36]. For instance, our previous studies demonstrated that low-dose genistein exposure attenuated the cancer-preventing effects of tamoxifen in cell line and mouse models of ErbB2-overexpressing breast cancer [34, 37]. Yet, specific factors that mediate ER-RTK crosstalk may vary under different environmental conditions and require further investigation. Despite preclinical data indicating that BPAF stimulates ER signaling and transcriptional activity, the impact of BPAF exposure on RTK signaling has not been reported. Thus, investigation of the effects of BPAF on ER-RTK crosstalk and functional consequences in context with ER^+^ breast carcinogenesis is necessary.

Overall, despite critical *in vitro* data highlighting the pro-estrogenic and endocrine-disrupting activities of BPAF, the specific molecular pathways associated with BPAF-mediated cellular responses remain uncertain. In our current study, we characterize the effect of BPAF on ER^+^ breast cancer cell lines and demonstrate the essential role of ER-RTK crosstalk in BPAF-induced cellular responses. Particularly, we identify AREG as a critical mediator of BPAF-induced ER-RTK crosstalk in ER^+^ breast cancer cells. These novel findings lay a solid foundation for future preclinical/clinical studies to determine the consequences of BPAF exposure on human ER^+^ breast cancer risk, which will have significant implications on the application of BPAF as a BPA alternative.

## Results

### BPAF promotes MCF-7 and T47D ER^+^ breast cancer cell proliferation and migration

**Fig 1:**
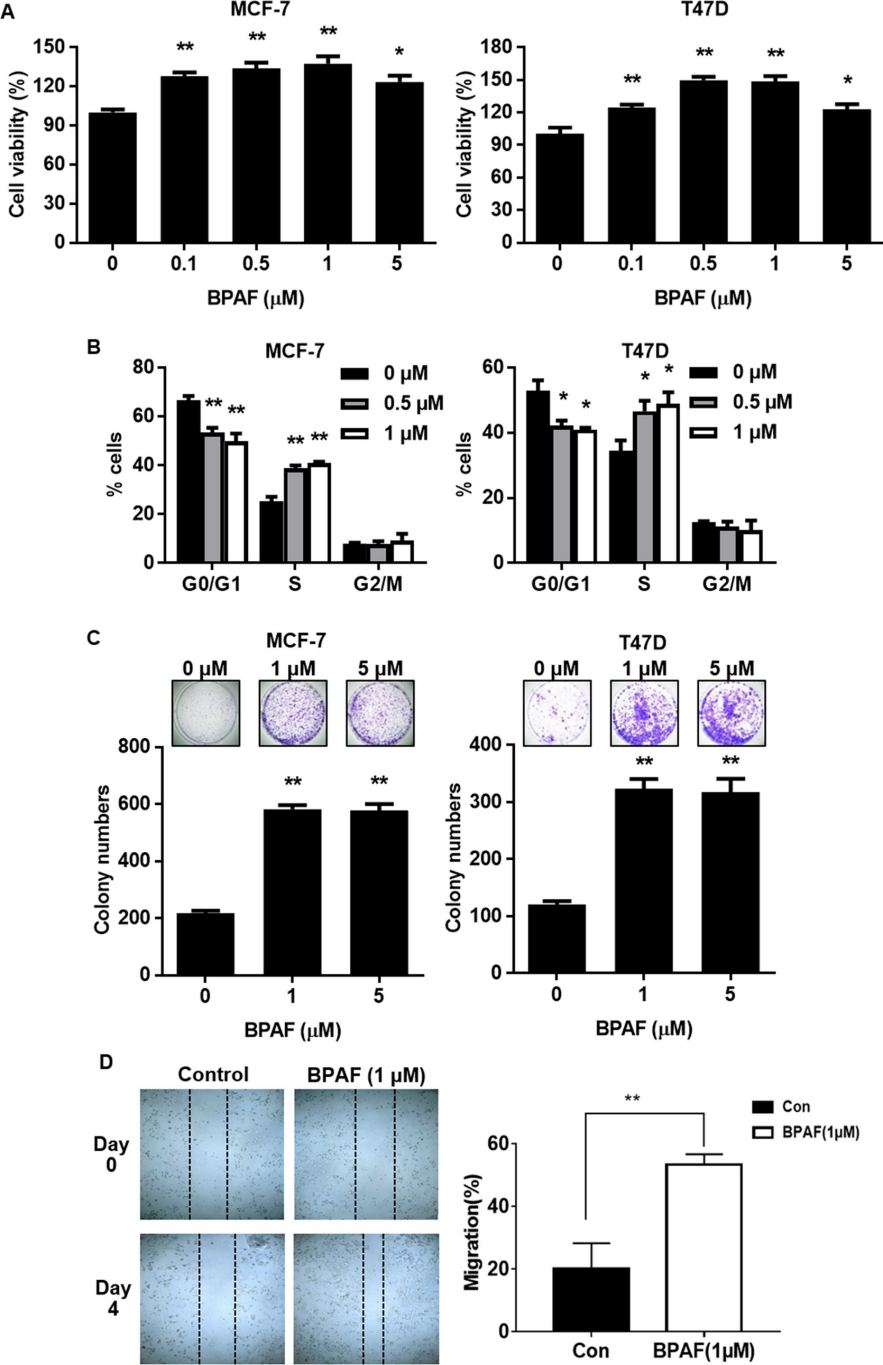
BPAF promotes ER^+^ breast cancer cell proliferation and migration. **A)** MCF-7 and T47D ER^+^ breast cancer cells were serum-starved for 48 hours in phenol red-free medium. Then, cells were treated with BPAF (0, 0.1, 0.5, 1, or 5 μM) in phenol red-free medium with 5% C.S. FBS for 5 days. The percentage of viable cells in each cell line was determined with an MTT assay. **B)** MCF-7 and T47D cells were serum-starved for 48 hours in phenol red-free medium. Then, cells were treated with BPAF (0, 0.5, or 1 μM) in phenol red-free medium with 5% C.S. FBS for 24 hours, followed by FACS analysis of the percentage of cells in G0/G1, S, and G2/M phases of the cell cycle. The average percentages of cells in each phase are graphed. **C)** MCF-7 and T47D cells were serum-starved for 48 hours in phenol red-free medium. Then, cells were treated with BPAF (0, 1, or 5 μM) in phenol red-free medium with 5% C.S. FBS for 21 days. Then, the cells were fixed and stained with crystal violet. The graphs in the lower panels present the average number of colonies formed with representative images in the panels above. **D)** The migration of cells treated with BPAF (0 or 1 μM) for 24 hours was determined by a wound healing assay. The panel to the left shows MCF-7 cells at Day 0 and Day 4 after the initial wound was formed. Representative images were captured at 10× magnification and dashed lines indicate the wound boundaries. The panel to the right depicts the percent of the wound width that the cells migrated after 4 days. All values are presented as the means ± standard error of the mean (S.E.) (*P<0.05, **P<0.01 as compared to the corresponding controls).

Previous reports have demonstrated that BPA and other bisphenols have a high affinity for ERs. Conditions for BPAF as an ER agonist and antagonist in ER^+^ breast cancer cells have not been well-established. Therefore, we first characterized BPAF-induced effects on ER^+^ breast cancer cells. Using an MTT assay, we found that low-dose BPAF (0.1–5 μM) significantly promoted cell proliferation in MCF-7 and T47D ER^+^ breast cancer cells (Fig 1A). Consistently, cell cycle analysis of MCF-7 and T47D cells indicated that low concentrations of BPAF (0.5 and 1 μM) significantly increased the proliferative population of cells in S phase, which was accompanied by a reduction in the percentage of cells in G0/G1 phase (Fig 1B). The proliferative effects of BPAF were further substantiated with a clonogenic assay, as demonstrated by a BPAF-induced 3-fold increase in colony numbers in both cell lines (Fig 1C). BPAF (1 μM) also markedly promoted MCF-7 cell migration as demonstrated by a wound healing assay (Fig 1D). Together, our data demonstrate the growth-promoting effects of low-dose BPAF on ER^+^ breast cancer cells.

### BPAF stimulates ER signaling in ER^+^ breast cancer cells

**Fig 2:**
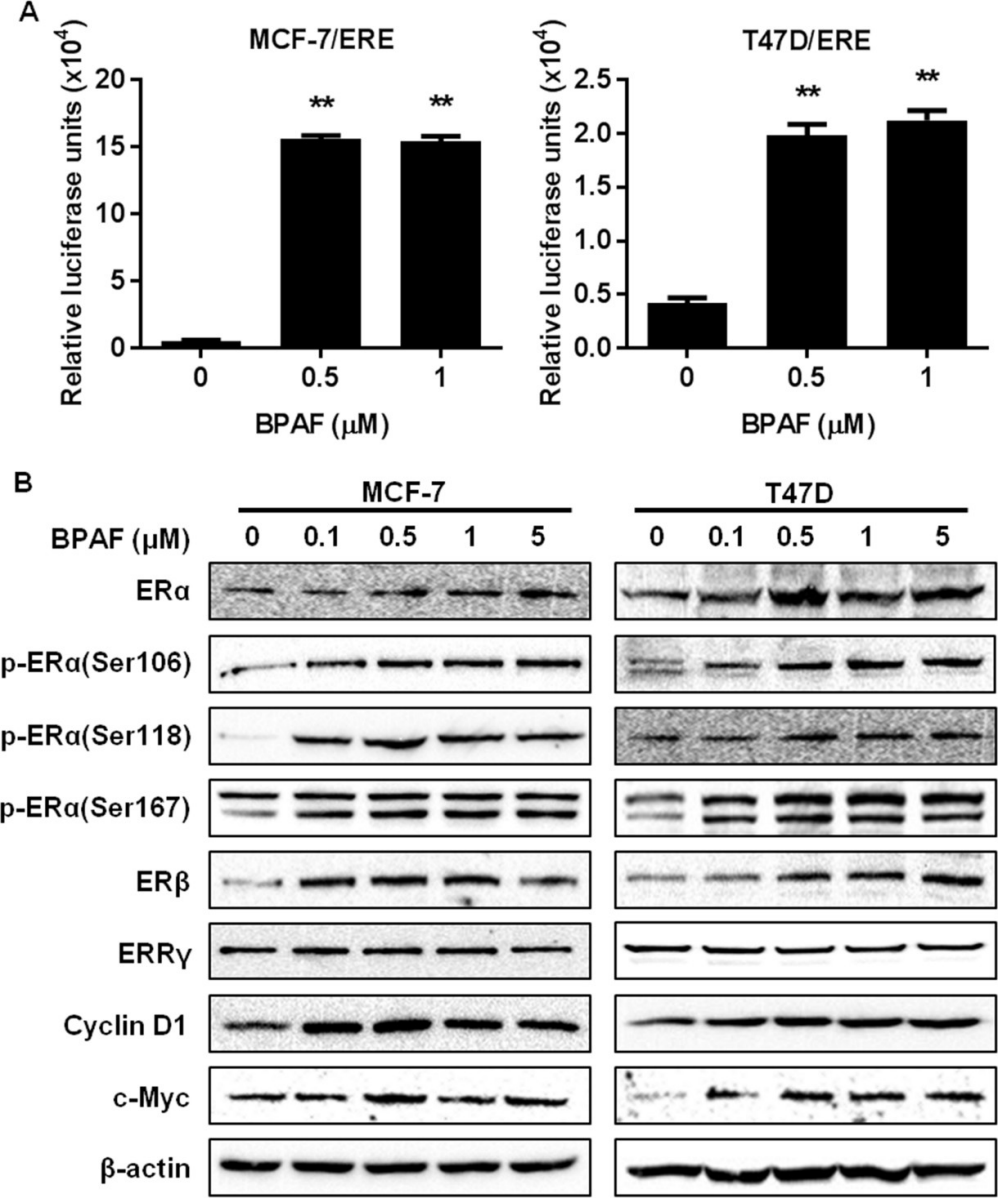
BPAF stimulates ER signaling. **A)** MCF-7 and T47D cells transiently transfected with the ERE luciferase reporter plasmid (MCF-7/ERE and T47D/ERE cells) were serum-starved for 48 hours in phenol red-free medium. Then, cells were treated with BPAF (0, 0.5, or 1 μM) in phenol red-free medium with 5% C.S. FBS for 24 hours. Cell lysates were used for the reporter assays to quantify the relative luciferase activities after each treatment. Values are presented as the means ± S.E. (**P<0.01). **B)** MCF-7 and T47D cells were serum-starved for 48 hours in phenol red-free medium. Then, cells were treated with BPAF (0, 0.1, 0.5, 1 or 5 μM) in phenol red-free medium with 5% C.S. FBS for 30 minutes, followed by Western blotting analysis of the indicated markers involved in the ER signaling pathways.

Since BPA is an EDC, we aimed to determine the endocrine-disrupting properties of BPAF in MCF-7 and T47D ER^+^ breast cancer cells. To this end, we used a luciferase reporter assay in MCF-7 and T47D cells transfected with ERE luciferase reporter plasmids (MCF-7/ERE and T47D/ERE cells) to examine the effects of BPAF on ER-mediated transcriptional activity. BPAF at 0.5 and 1 μM significantly increased in luciferase activity in both cell lines (Fig 2A), indicating the promotion of ER-mediated transcriptional activity. BPAF-induced transcriptional regulation of the ER pathway was also accompanied by a dose-dependent increase in ERα protein expression and activation/phosphorylation (Fig 2B). As well, ERβ and downstream effectors of ER signaling, including Cyclin D1 and c-Myc, were upregulated by BPAF. Although it was reported that ERRγ is a cellular target of BPA [38, 39], we found that BPAF did not affect ERRγ expression, which can be justified by the low binding affinity of BPAF for ERRγ [11]. The BPAF-mediated gene and protein regulation of the ER signaling pathway supports BPAF as a potent ER agonist at low concentrations.

### BPAF induces growth factor/RTK signaling in ER^+^ breast cancer cells

**Fig 3:**
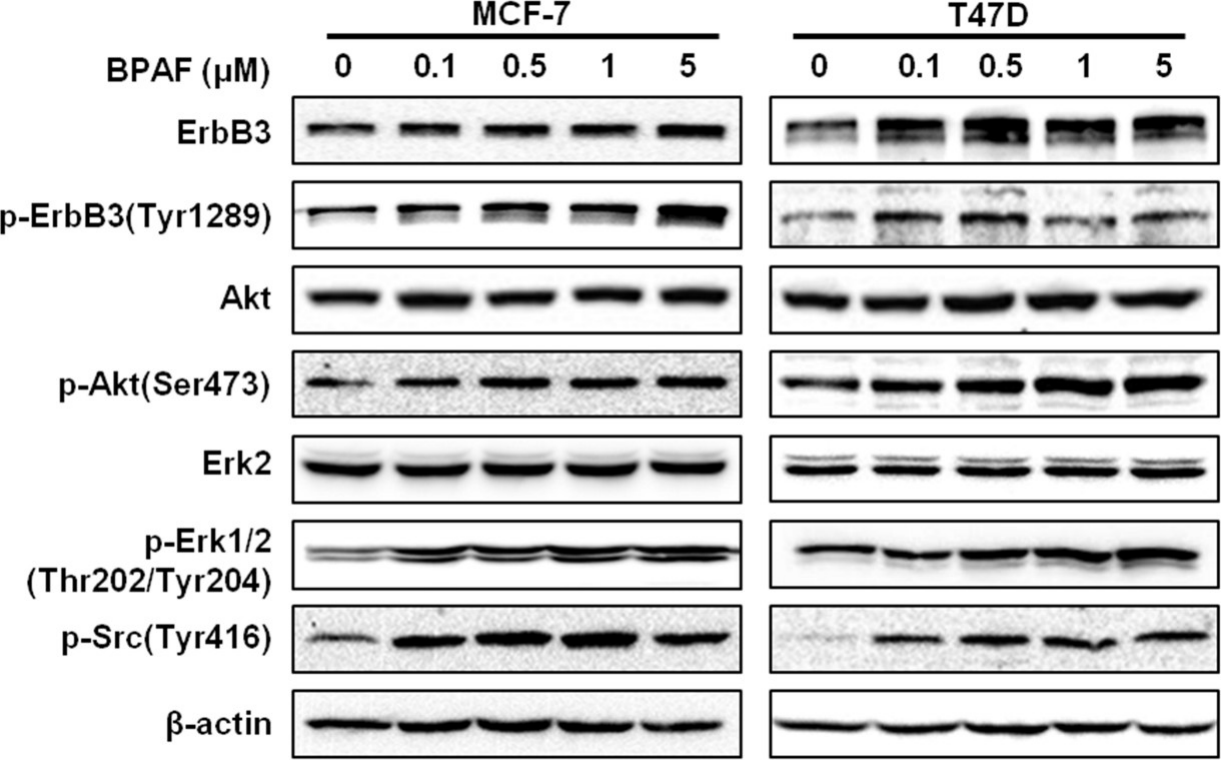
BPAF stimulates ErbB3/RTK signaling. MCF-7 and T47D cells were serum-starved for 48 hours in phenol red-free medium. Then, cells were treated with BPAF (0, 0.1, 0.5, 1 or 5 μM) in phenol red-free medium with 5% C.S. FBS for 30 minutes, followed by Western blotting analysis of the indicated markers involved in the ErbB3/RTK signaling pathways.

To further investigate the effects of BPAF on molecular signaling in ER^+^ breast cancer cells, we examined the expression and activation/phosphorylation of key markers involved in the RTK signaling cascade in BPAF-exposed cells. As shown in Fig 3, BPAF (0–5 μM) induced ErbB3 expression and activation/phosphorylation, and also phosphorylated Akt and Erk1/2, indicating the activation of downstream signaling in the PI3K/Akt and MAPK/Erk pathways. In context with BPAF-induced activation of ER signaling, concurrent activation of both pathways strongly supports that BPAF promotes crosstalk between the ER and RTK signaling pathways, which may amplify BPAF-induced cell proliferation (Fig 1).

### Disruption of the ER pathway blocks BPAF-induced cell proliferation and ER-dependent cellular responses

**Fig 4:**
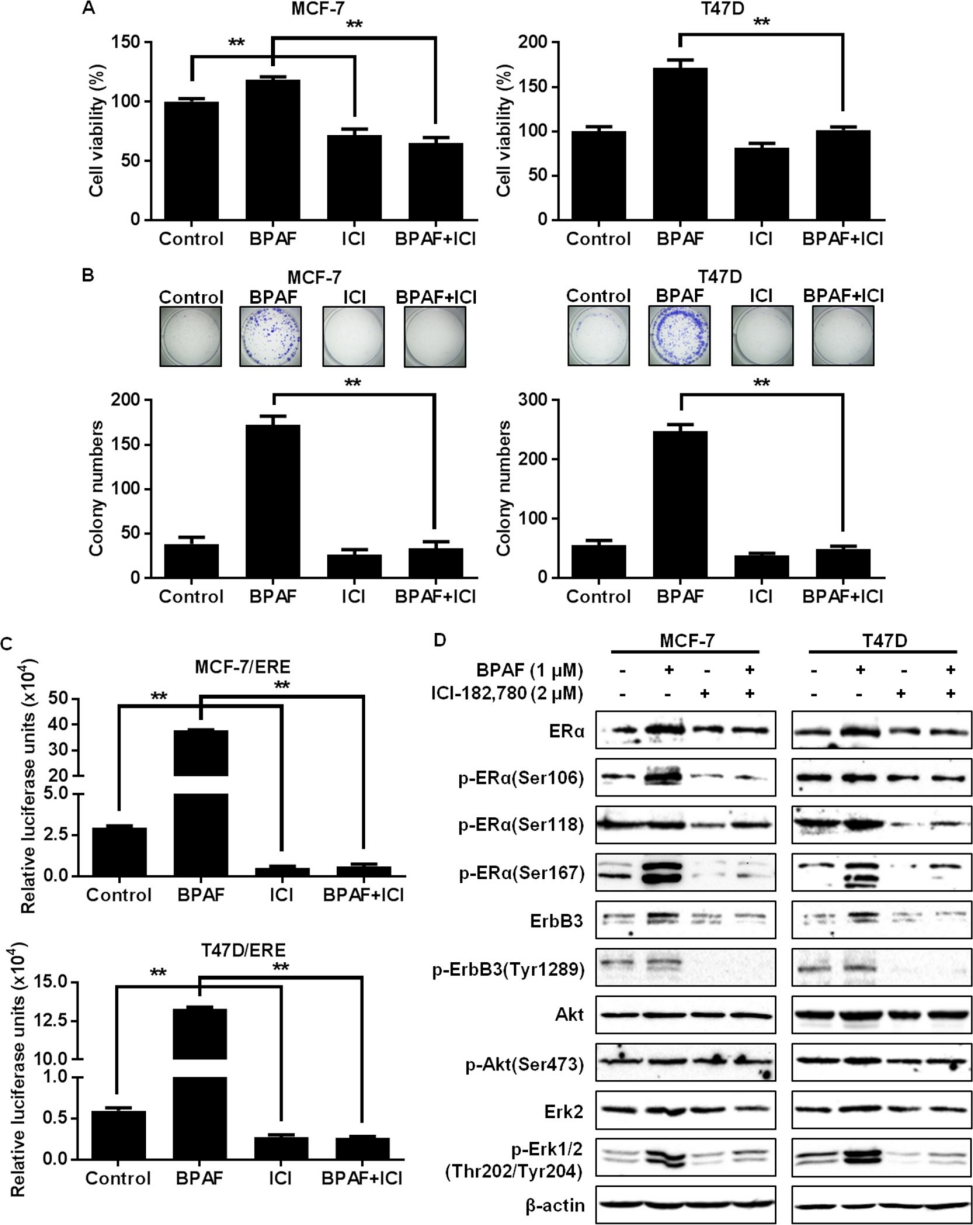
Disruption of the ER pathway blocks BPAF-induced cell growth and ER-mediated transcriptional activity. **A)** MCF-7 and T47D cells were serum-starved for 48 hours in phenol red-free medium. Then, cells were treated with BPAF (1 μM) ± ICI-182,780 (2 μM) in phenol red-free medium with 5% C.S. FBS for 5 days. The average percentage of viable cells in each treatment group was determined with an MTT assay. **B)** MCF-7 and T47D cells were serum-starved for 48 hours in phenol red-free medium. Then, cells were treated with BPAF (1 μM) ± ICI-182,780 (2 μM) in phenol red-free medium with 5% C.S. FBS for 21 days, followed by fixation and staining with crystal violet. The graphs in the lower panels present the average number of colonies formed with representative images in the panels above. **C)** MCF-7/ERE and T47D/ERE cells transiently transfected with ERE luciferase reporters were serum-starved for 48 hours in phenol red-free medium. Then, cells were treated with BPAF (1 μM) ± ICI-182,780 (2 μM) in phenol red-free medium with 5% C.S. FBS for 24 hours. The relative luciferase activities for each treatment group are graphed. All values are presented as the means ± S.E. (**P<0.01). **D)** MCF-7 and T47D cells were serum-starved for 48 hours in phenol red-free medium. Then, cells were pretreated with ICI-182,780 (2 μM) in phenol red-free medium with 5% C.S. FBS for 16 hours, followed by treatment with BPAF (1 μM) in phenol red-free medium with 5% C.S. FBS for 30 minutes. Western blotting analysis was performed on the indicated markers involved in the ER and ErbB3/RTK signaling pathways.

To understand the role that ER signaling plays in BPAF-mediated cellular responses, we blocked ER signaling using the selective ER downregulator (SERD)/ERα antagonist, ICI-182,780. In turn, we determined that ICI-182,780 treatment remarkably attenuated BPAF-induced cell proliferation, as demonstrated by MTT and colony formation assays (Fig 4A and 4B). As well, results from luciferase reporter assays demonstrated that ERE-mediated transcriptional activity was significantly downregulated in cells treated with BPAF (1 μM) + ICI-182,780 (2 μM) as compared to BPAF alone (Fig 4C). Importantly, ER blockage by ICI-182,780 also suppressed the BPAF-induced activation/phosphorylation of ERα, ErbB3, Akt, and Erk1/2 (Fig 4D). These data provide critical evidence that ER signaling is not only essential for BPAF-mediated ER pathway activation, but also is critical for BPAF-induced growth factor/RTK pathway activation in ER^+^ breast cancer cells.

### Inhibition of EGFR and PI3K blocks BPAF-induced cell proliferation

**Fig 5:**
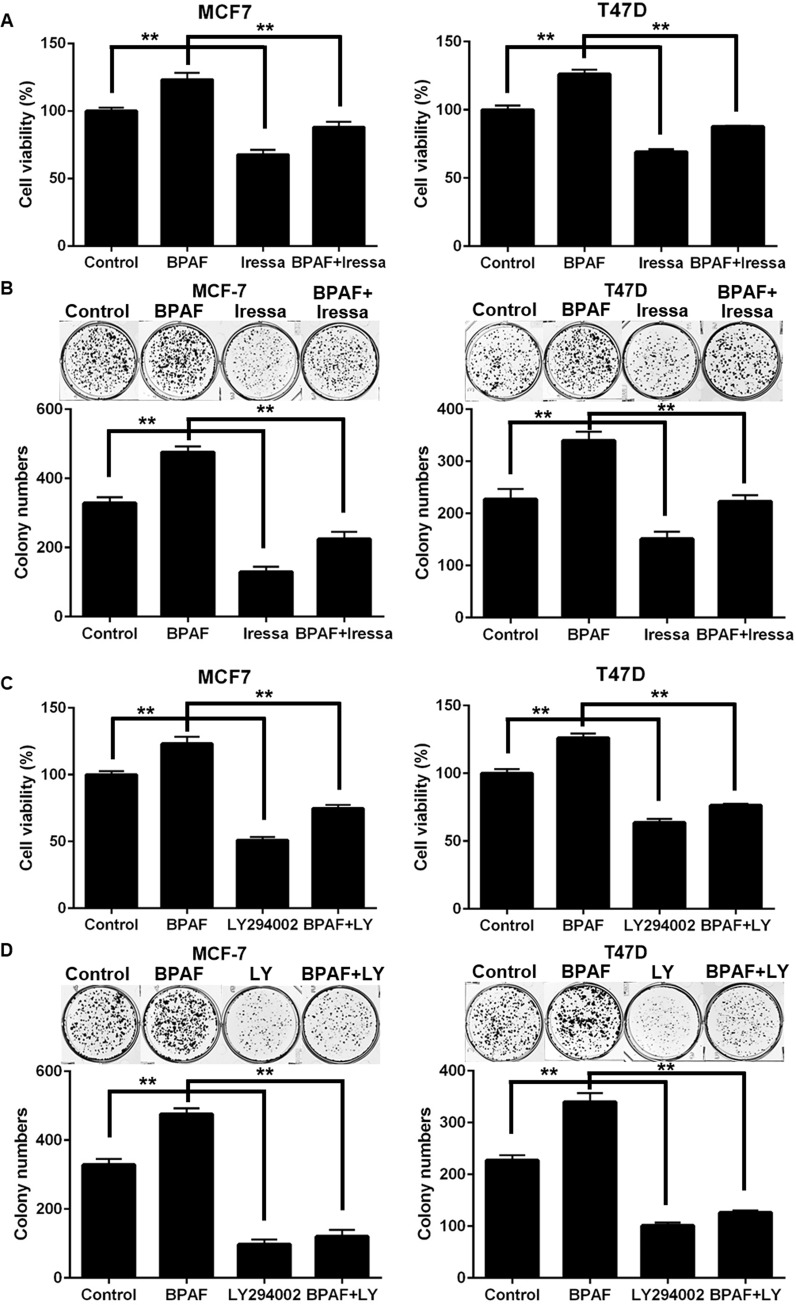
Inhibition of EGFR and PI3K blocks BPAF-induced cell growth. **A)** MCF-7 and T47D cells were serum-starved for 48 hours in phenol red-free medium. Then, cells were treated with BPAF (1 μM) ± Iressa (2 μM) in phenol red-free medium with 5% C.S. FBS for 5 days. The average percentage of viable cells in each treatment group was determined with an MTT assay. **B)** MCF-7 and T47D cells were serum-starved for 48 hours in phenol red-free medium. Then, cells were treated with BPAF (1 μM) ± Iressa (2 μM) in phenol red-free medium with 5% C.S. FBS for 21 days, followed by fixation and staining with crystal violet. The graphs in the lower panels present the average number of colonies formed with representative images in the panels above. **C)** MCF-7 and T47D cells were serum-starved for 48 hours in phenol red-free medium. Then, cells were treated with BPAF (1 μM) ± LY294002 (5 μM) in phenol red-free medium with 5% C.S. FBS for 5 days. The average percentage of viable cells in each treatment group was determined with an MTT assay. **D)** MCF-7 and T47D cells were serum-starved for 48 hours in phenol red-free medium. Then, cells were treated with BPAF (1 μM) ± LY294002 (5 μM) in phenol red-free medium with 5% C.S. FBS for 21 days, followed by fixation and staining with crystal violet. The graphs in the lower panels present the average number of colonies formed with representative images in the panels above. All values are presented as the means ± S.E. (**P<0.01).

Previous studies have reported that BPA can activate RTKs, including EGFR [40, 41]. However, limited data are available in regard to the oncogenic effects of BPAF and EGFR signaling. Therefore, in order to determine whether EGFR signaling contributes to BPAF-mediated cell proliferation, we suppressed EGFR signaling via treatment with the selective EGFR inhibitor, Iressa (Gefitinib). Our data demonstrated that treatment with Iressa significantly blocked BPAF-induced cell proliferation in MCF-7 and T47D ER^+^ breast cancer cells, as determined by MTT and colony formation assays (Fig 5A and 5B). Furthermore, we examined the role of downstream PI3K in BPAF-induced cellular responses. As such, we blocked PI3K signaling with the selective PI3K inhibitor, LY294002, and found that PI3K inhibition significantly abolished BPAF-induced cell growth and colony formation (Fig 5C and 5D). Similarly, Ptak *et al*. found that PI3K inhibition via LY294002 significantly abrogated BPA-induced oncogenic responses *in vitro* [42]. Together, these data further indicate the potential RTK and downstream pathways that are critical for BPAF-induced oncogenic responses in ER^+^ breast cancer.

### AREG is a sensitive target of BPAF in ER^+^ breast cancer cells

**Fig 6:**
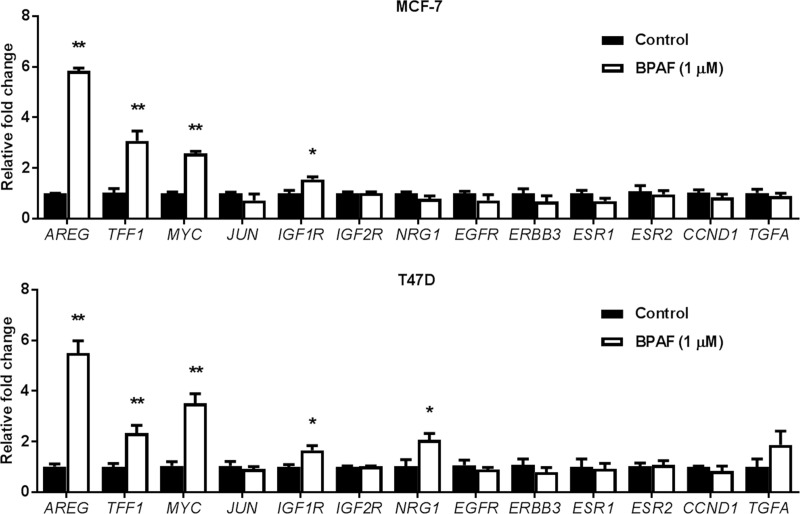
BPAF upregulates the expression of ER and growth factor target genes. MCF-7 and T47D cells were serum-starved for 48 hours in phenol red-free medium. Then, cells were treated with BPAF (0 or 1 μM) in phenol red-free medium with 5% C.S. FBS for 16 hours, followed by qPCR analysis of the indicated ER and growth factor gene targets. The fold changes for the BPAF-treated samples are graphed relative to the normalized values of the corresponding controls. Values are presented as the means ± S.E. (*P<0.05, **P<0.01 as compared to the corresponding controls).

Given the marked ER and RTK signaling promotion induced by BPAF, we determined the BPAF-mediated changes in mRNA levels of key regulators of the ER and RTK pathways, including *AREG*, *NRG1*, *IGF1R*, *IGF2R*, *EGFR*, *ERBB3*, *ESR1*, *ESR2*, *CCND1*, *TFF1*, *MYC*, *JUN*, and *TGFA*. We found that *AREG*, *TFF1*, *MYC*, and *IGF1R* mRNA levels were significantly upregulated by BPAF in both MCF-7 and T47D cells (Fig 6). To note, BPAF stimulated a more than 5-fold increase in the mRNA expression of *AREG* in both cell lines. Together with concurrent BPAF-induced activation of ER and RTK signaling pathways, our qPCR data demonstrated that the upregulation of growth factors/ligands is a component of BPAF-induced ER-RTK crosstalk. As *AREG* is an ER-targeted gene that encodes for the RTK ligand protein amphiregulin [43], our finding that BPAF potently upregulates *AREG* in ER^+^ breast cancer cells is novel.

### AREG is a critical mediator of BPAF-induced ER-RTK signaling crosstalk

**Fig 7:**
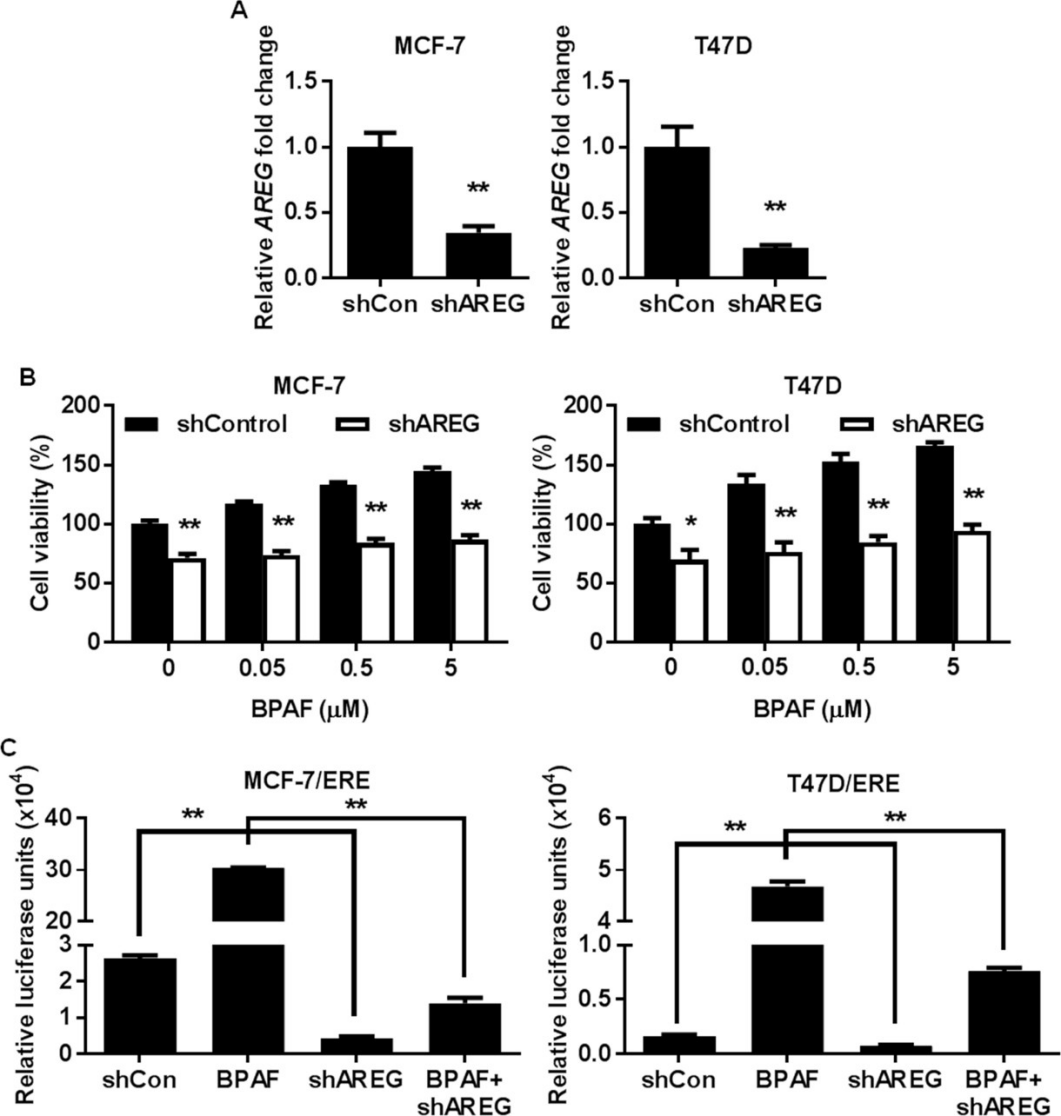
AREG knockdown impedes BPAF-mediated cell proliferation and ER transcriptional activity. MCF-7 and T47D cells were transiently transfected with lentivirus-mediated AREG shRNA. PCR validation of AREG knockdown is shown in **A**. **B)** Control and AREG shRNA MCF-7 and T47D cells were serum-starved for 48 hours in phenol red-free medium. Then, cells were treated with BPAF (0, 0.05, 0.5, or 5 μM) in phenol red-free medium with 5% C.S. FBS for 5 days. The average percentage of viable cells in each treatment group was determined with an MTT assay. **C)** Control and AREG shRNA MCF-7 and T47D cells were transiently transfected with the ERE luciferase reporter plasmids, followed by serum starvation in phenol red-free medium for 48 hours. Then, the cells were exposed to BPAF (1 μM) in phenol red-free medium with 5% C.S. FBS for 24 hours. The relative luciferase activities for each treatment group are graphed. All values are presented as the means ± S.E. (**P<0.01).

**Fig 8:**
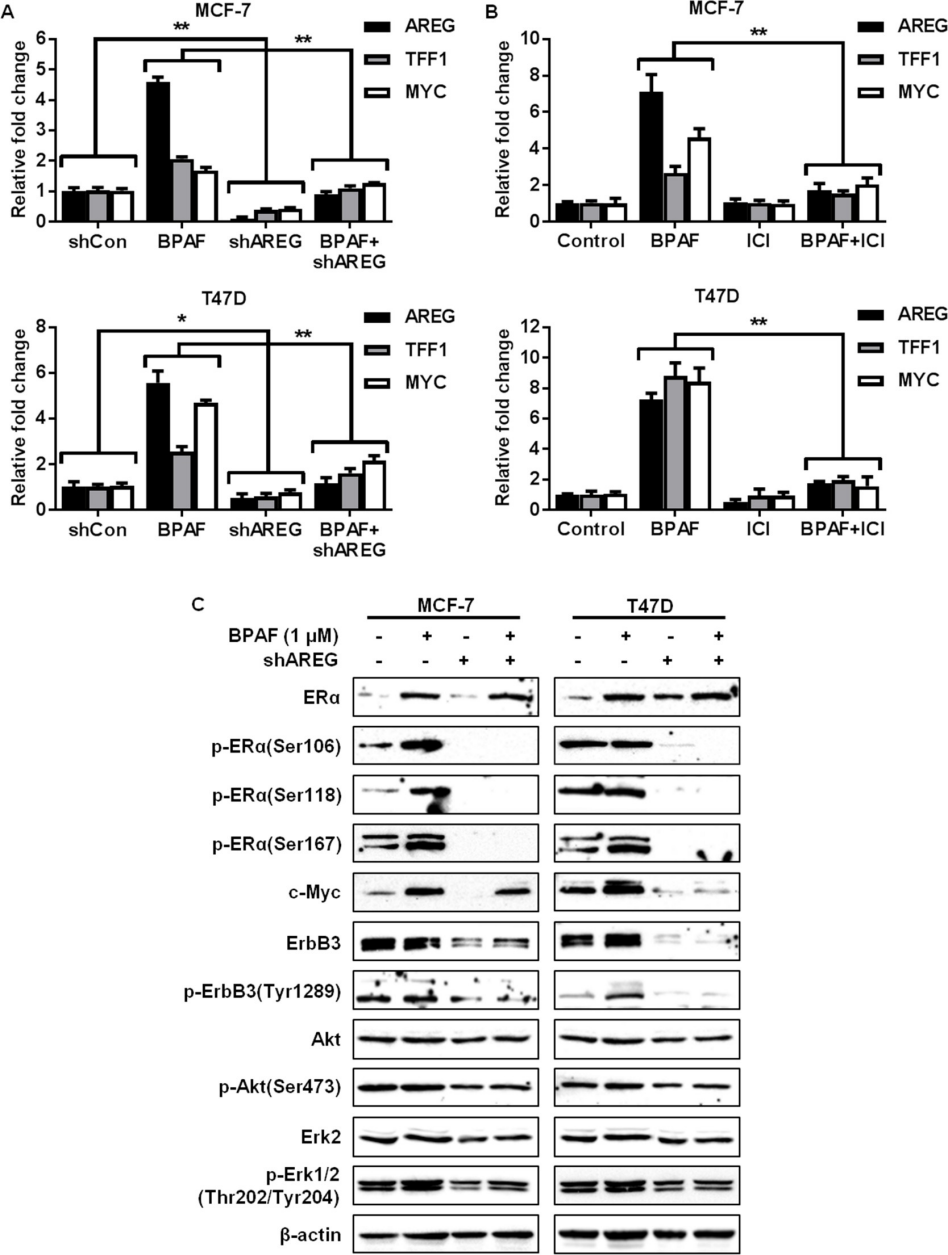
AREG knockdown blocks BPAF-mediated ER-RTK crosstalk. Control and AREG shRNA MCF-7 and T47D cells were serum-starved for 48 hours in phenol red-free medium. Then, cells were treated with BPAF (0 or 1 μM) in phenol red-free medium with 5% C.S. FBS for 16 hours (**A**) and MCF-7 and T47D cells were treated with BPAF (1 μM) ± ICI-182,780 (2 μM) in phenol red-free medium with 5% C.S. FBS for 16 hours (**B**), followed by qPCR analysis of *AREG*, *TFF1*, and *MYC*, the ER/RTK target genes most significantly upregulated by BPAF. The fold changes for the treated samples are graphed relative to the normalized values of the corresponding controls. Values are presented as the means ± S.E. (*P<0.05, **P<0.01 as compared to the corresponding treatment groups). **C)** Control and AREG shRNA MCF-7 and T47D cells were serum-starved for 48 hours in phenol red-free medium. Then, cells were treated with BPAF (0 or 1 μM) in phenol red-free medium with 5% C.S. FBS for 30 minutes. Then, Western blotting analysis was performed on the indicated markers involved in the ER and ErbB3/RTK signaling pathways.

Previous studies have reported that AREG expression is upregulated by estrogen both *in vitro* and *in vivo*, and that high *AREG* expression is more prevalent in human ER^+^ breast cancers than ER^-^ breast cancers [43–46], suggesting that AREG upregulation may be associated with increased ER^+^ breast cancer risk. However, the specific connection between BPAF-induced AREG expression and ER^+^ breast cancer risk has not been investigated. Therefore, to determine the importance of AREG in BPAF-induced cellular responses and ER-RTK crosstalk, we knocked down *AREG* via lentivirus-mediated shRNA (Fig 7A) and examined the effects on BPAF-mediated cell growth and transcriptional regulation. To this end, in contrast to BPAF-induced cell proliferation in the control cells infected with control lentiviruses (shControl), AREG knockdown (shAREG) abrogated the effects of BPAF on ER^+^ breast cancer cell growth (Fig 7B). AREG shRNA also significantly impeded BPAF-induced ER-mediated transcriptional activity in MCF-7/ERE and T47D/ERE cells, as indicated by luciferase reporter assays (Fig 7C). Consistently, AREG knockdown blocked BPAF-mediated upregulation of *AREG*, *TFF1*, and *MYC* mRNA expression (Fig 8A). These findings were also consistent with the ICI-182,780-mediated downregulation of *AREG*, *TFF1*, and *MYC* (Fig 8B) [18]. AREG is an important mediator of ER and RTK signaling [43–45]; therefore, we further analyzed the BPAF-induced changes in protein expression and phosphorylation/activation associated with the ER and RTK pathways in control and AREG knockdown cells. Importantly, our results demonstrate that AREG knockdown blocks BPAF-induced ERα, ErbB3, Akt, and Erk1/2 activation/phosphorylation, and ErbB3 expression, as compared to BPAF-exposed MCF-7 and T47D cells with endogenous AREG expression (Fig 8C). Since AREG knockdown not only suppressed RTK signaling, but also suppressed ER-mediated transcription and protein signaling, our data confirm that AREG is an essential mediator of BPAF-induced signaling interactions between the ER and RTK pathways. These findings provide a critical link between BPAF exposure and ER-RTK crosstalk-mediated ER^+^ breast carcinogenesis.

## Discussion

In our current study, we aimed to characterize the estrogenic and cancer-promoting effects of BPAF using ER^+^ breast cancer cells. We demonstrated that BPAF has significant growth promoting effects on ER^+^ breast cancer cells. As well, RTK signaling activation is critical for these BPAF-mediated cellular responses, which has not been previously reported. Furthermore, we identified ER-RTK crosstalk as an underlying mechanism that promotes BPAF-induced ER^+^ breast cancer cell proliferation. Importantly, our major novel finding revealed that AREG is a critical mediator of BPAF-induced ER-RTK crosstalk and is essential for the cancer-promoting effects of BPAF in our *in vitro* cell line models of ER^+^ breast cancer.

As a BPA analog, the endocrine-disrupting and related effects of BPAF have been tested in comparison with BPA using various preclinical models. Although multiple labs have demonstrated that BPAF exhibits potent binding affinities for ERα, ERβ, and GPER [11–13], most data are derived from biochemical experiments that do not reflect physiological exposure conditions. Thus, further studies are necessary to advance our understanding of the endocrine-disrupting consequences of BPAF and the implications associated with human diseases. To this end, our current study provides an in depth mechanistic investigation of the underlying mechanisms associated with BPAF-mediated cellular responses and the particular role of BPAF-induced ER-RTK signaling crosstalk in ER^+^ breast cancer cells. As such, we show a biphasic trend in which BPAF at concentrations less than 5 μM promote cell growth, as demonstrated by MTT, cell cycle, and clonogenic analyses (Fig 1). This finding is consistent with studies by other researchers reporting that higher concentrations of BPAF (greater than 10 μM) inhibited cell viability in MCF-7 ER^+^ breast cancer cells [47, 48]. Moreover, the BPAF-mediated phenotypic effects associated with cell proliferation were accompanied by data from our ERE-luciferase reporter, ER signaling, and ER-targeted gene expression assays, which collectively support the estrogenic/ERα agonist activities of BPAF. Together, these data confirm BPAF as a potent endocrine disruptor and potential promoter of ER^+^ breast cancer risk.

Although previous preclinical studies support the ERα agonist activities of BPAF, data are limited regarding the specific mechanisms that contribute to BPAF-mediated ER signaling and transcriptional activation. Given this gap in the current literature, we significantly advanced our understanding by examining the effects of BPAF on RTK signaling in ER^+^ breast cancer cells. Indeed, we found that BPAF stimulated concurrent activation of ER and RTK signaling pathways, as demonstrated by the phosphorylation/activation of downstream PI3K/Akt and MAPK/Erk. Our novel data further implicated PI3K signaling in BPAF-mediated cellular responses, as shown by the significant inhibition of BPAF-mediated cell proliferation upon treatment with the selective PI3K inhibitor LY294002 (Fig 5). Future studies are warranted to examine the role of PI3K kinase activity [49] and downstream PI3K/Akt signaling in the proliferative effects of BPAF in ER^+^ breast cancer cells. In particular, Akt and Erk have been shown to phosphorylate ERα at Ser167, as well as activate the ERα estrogen-independent domain, AF-1 [50]. Furthermore, Li *et al*. found that Erk1/2 activation was required for ER-mediated transcriptional activity [18]. Consistent with these data, we found that BPAF was potent enough to phosphorylate ERα at Ser167 and Erk1/2. As well, treatment with the ERα antagonist ICI-182,780 abrogated both the ER and RTK signaling pathways, which further supports the induction of ER-RTK crosstalk in BPAF-exposed ER^+^ breast cancer cells. Our findings not only identify ER-RTK crosstalk as a major underlying mechanism of BPAF-induced cellular responses, but may also provide novel diagnostic markers for women at risk for ER^+^ breast cancer.

Another of our major findings is the identification of AREG as a sensitive target and critical mediator of BPAF-induced cellular responses. AREG is a ligand specific for EGFR, which can form dimers with and transactivate other EGFR/ErbB family members, such as ErbB2 and ErbB3 [51]. Several studies have also implicated AREG in ERα-mediated mammary gland development and cancer development [43–46]. In particular, Peterson *et al*. determined that high AREG expression correlates with increased tumor multiplicity in ER^+^ breast cancer patients [43]. Since ERα activation can promote the expression of growth factors and other ligands, such as TGFα, IGF1, and NRG, different exposure conditions may elicit the stimulation of different responses. Nevertheless, given that ICI-182,780 treatment blocks BPAF-induced AREG, our study underscores AREG as a sensitive downstream effector of BPAF-induced ERα activation. In conjunction with previous studies, our data highlight AREG as a potential therapeutic target for BPAF-associated ER^+^ breast cancer.

In the current study, we focused on the genomic activation of ERα by BPAF. Future studies will investigate the role of non-genomic ERα activation, as well as other pathways that may be involved in BPAF-associated ER^+^ breast cancer cellular responses. In particular, the activation of multiple RTKs, including EGFR, and their downstream signaling may serve a complex role in BPAF-mediated oncogenic responses, which requires further investigation. As such, Bilancio *et al*. reported anti-proliferative effects of BPA, including EGFR/Erk-dependent cell cycle arrest and p53 phosphorylation at Ser15, in prostate cancer cell models [40]. Similarly, others have found a correlation between non-genomic estrogen signaling via GPR30 and the subsequent phosphorylation of p53 at Ser15 and cell cycle arrest in ER^-^ breast cancer cells [52]. Given these intriguing findings, the role of EGFR and downstream signaling pathways, such as PI3K/Akt and MAPK/Erk, in BPAF-mediated cellular responses in ER^+^ breast cancer cell models needs to be further examined. Importantly, our current study provides essential preclinical mechanistic data that substantiates the endocrine-disrupting activities of BPAF and the potential underlying mechanisms involving the activation of ER-RTK crosstalk that may contribute to the promotion of ER^+^ breast carcinogenesis. Overall, given the potent effects of BPAF on cellular phenotypes and signaling in ER^+^ breast cancer cells *in vitro*, our current study ultimately provides substantial evidence warranting future preclinical and clinical studies that will advance our understanding of BPAF exposure conditions and assess the safety of BPAF as an alternative to BPA.

## Materials and methods

### Antibodies and reagents

BPAF was purchased from Sigma (St. Louis, MO). LY294002 (PI3K inhibitor) and Iressa (Gefitinib) were purchased from LC Laboratories (Woburn, MA). Primary antibodies against ERα, phosphorylated-ERα (p-ERα) (Ser106), ERβ, ERRγ, Akt, Erk2, and β-actin were purchased from Santa Cruz Biotechnology (Santa Cruz, CA). p-ERα (Ser118), Cyclin D1, c-Myc, ErbB3, p-ErbB3 (Tyr1289), p-Akt (Ser473), p-Erk1/2 (Thr202/Tyr204), and p-Src (Tyr416) primary antibodies were purchased from Cell Signaling (Danvers, MA). The primary antibody against p-ERα (Ser167) was purchased from Thermo Fisher Scientific (Rockford, IL).

### Cell culture

MCF-7 and T47D human breast cancer cell lines were purchased from the American Type Culture Collection (ATCC; Manassas, VA). All cells were maintained in DMEM/F12 culture medium supplemented with 10% FBS, 100 μg/mL penicillin, and 100 μg/mL streptomycin at 37°C in an incubator with a humidified 5% CO_2_ atmosphere. Prior to control and BPAF treatments, cells were serum-starved for 48 hours in phenol red-free medium. Then, cells were treated with BPAF in phenol red-free DMEM/F12 with 5% charcoal:dextran-stripped (C.S.) FBS (Gemini Bio-Products; Sacramento, CA) for the indicated treatment durations.

### Cell proliferation assay

Cells were seeded (2×10^3^ cells/well in complete medium) in 96-well plates for 24 hours. Then, after serum starvation for 48 hours, the cells were treated with BPAF in phenol red-free DMEM/F12 medium with 5% C.S. FBS for 5 days. On the 5^th^ day, cells were incubated in 50 μL MTT solution (2.5 mg/mL) for 4 hours. Next, the absorbance at 540 nm was quantified with a SynergyMx microplate reader (BioTek; Winooski, VT) to calculate the percentage of viable cells.

### Cell cycle analysis

Cells were seeded (1×10^4^ cells/plate in complete medium) in 60 mm plates for 24 hours. Following serum starvation for 48 hours, the cells were treated with BPAF in phenol red-free DMEM/F12 medium with 5% C.S. FBS for 24 hours. The collected cells were fixed in 70% ethanol overnight at -20°C. After the fixed cells were washed in PBS, the cells were incubated in PBS with RNase A (0.5 mg/mL) and propidium iodide (PI; 50 μg/mL) for 30 minutes at 37°C. The percentage of cells in each phase of the cell cycle was quantified using a Guava easyCyte 8 flow cytometer (Millipore; Billerica, MA) with ModFit software.

### Clonogenic assay

Cells were seeded (1×10^3^ cells/well in complete medium) in 6-well plates for 24 hours. Cells were serum-starved for 48 hours and then treated with BPAF in phenol red-free DMEM/F12 medium with 5% C.S. FBS for 21 days. Next, cells were stained with 0.5% crystal violet and colonies with ≥ 50 cells were counted for each sample. Images of the stained colonies were captured with a Nikon SMZ 745T microscope and Nikon Elements Imaging System Software.

### Wound healing assay

Wound healing assays were performed as previously described [53]. Briefly, cells were seeded in 6-wells plates and cultured in serum-free phenol red-free DMEM/F12 medium until 90–100% confluence. Using a pipette tip, a wound was made in the monolayer of cells in each well. Debris was removed and cells were treated with BPAF in phenol red-free DMEM/F12 medium with 5% C.S. FBS for 24 hours. Then, cells were cultured for another 3 days. Images of the same wound at Day 0 and Day 4 after the wound was created were captured with a Nikon SMZ 745T microscope and Nikon Elements Imaging System Software (10x magnification). The percentage of migration was calculated as the difference between the wound width at Day 0 and Day 4. The wound width at Day 0 was normalized to 100%.

### Luciferase reporter assay

Cells were seeded in 12-well plates and incubated overnight. The cells were transfected using X-tremeGENE 9 DNA transfection reagent (Roche; Indianapolis, IN) according to the manufacturer’s instructions. The ERE-luciferase constructs used in our study were a kind gift from Dr. Donald P. McDonnell (Duke University). Briefly, the ERE luciferase plasmid DNA was transfected into the cells for 24 hours. Then, the cells were serum-starved for 48 hours, followed by BPAF treatment in phenol red-free DMEM/F12 medium with 5% C.S. FBS for 24 hours. The luciferase activity was measured using Luciferin Detection Reagent (Promega; Madison, WI) and a Modulus single tube reader (Turner BioSystems; Sunnyvale,CA).

### Western blot analysis

Protein concentrations of whole cell lysates were quantified using a BCA Protein Assay kit (Thermo Fisher Scientific). Equal amounts of protein (50 μg) were separated using 10% or 12% SDS-PAGE and were transferred to nitrocellulose membranes. After blocking in 5% non-fat milk for 2 hours at room temperature, the membranes were incubated in diluted primary antibodies overnight at 4°C. The following day, the membranes were washed in TBST buffer and incubated in horseradish peroxidase (HRP) secondary antibodies for 1.5 hours at room temperature. After final washes in TBST buffer, SuperSignal West Pico ECL solution (Thermo Fisher Scientific) was added to the membranes to enhance the chemiluminescent signal. Proteins bands were imaged using a FluorChemE imager.

### RNA isolation and real-time qPCR

RNA was extracted with TRIzol reagent (Life Technologies; Carlsbad, CA) following standard RNA extraction procedures. Total RNA (1μg) was reverse transcribed using MMLV reverse transcriptase (Bio-Rad; Hercules, CA) and the resulting cDNA was used for qPCR reactions with All-in-One qPCR Mix (GeneCopoeia). The triplicate samples were amplified in 20 μL reactions with gene-specific primers. The relative fold change of mRNA expression was determined for each group using the 2^-ΔΔCt^ method with *GAPDH* expression as an internal control.

### Lentiviral production and infection

The AREG shRNA-encoding lentiviral vector psi-LVRH1GH was purchased from GeneCopoeia (Rockville, MD). For lentivirus production, control or shRNA-encoding lentiviral vectors were transfected into 293T cells using the Lenti-Pac HIV Expression Packaging Kit (GeneCopoeia) according to the manufacturer’s instructions. The stocks of control and shRNA lentiviruses were collected at 24 and 48 hours after transfection and concentrated by ultracentrifugation. For viral infection, cells were seeded (1×10^6^ cells/plate) in 60 mm plates at 24 hours prior to infection. Then, the resulting AREG knockdown cells were serum-starved for 48 hours and treated with BPAF in phenol red-free DMEM/F12 medium with 5% C.S. FBS according to the indicated experimental procedures.

### Statistical analysis

The statistical differences between groups were evaluated by Student’s t-tests using GraphPad Prism software (La Jolla, CA). A *P*-value of 0.05 (*P*<0.05) was chosen for significance.
